# Case Report: *Shewanella algae*, a rare cause of osteosynthesis-associated infection

**DOI:** 10.12688/f1000research.142096.2

**Published:** 2024-09-02

**Authors:** Sofiane Masmoudi, Mohamed Ali Khlif, Hajer Battikh, Meriam Zribi, Maher Barsaoui, Khaled Zitouna

**Affiliations:** 1 University of Tunis El Manar, Tunis, Tunis, Tunisia; 2 Orthopedic surgery, Rabta Hospital, Tunis, Tunis, Tunisia; 3 Microbiology, Rabta Hospital, Tunis, Tunis, Tunisia

**Keywords:** Shewanella, Osteosynthesis, Infection, Osteosynthesis-associated infection, Hematogenous

## Abstract

*Shewanella* is an emerging human pathogen. It mostly causes skin and soft tissue infections. Osteosynthesis-associated infection involving *Shewanella* are rare and in most cases are secondary to direct contamination following open fractures in aquatic environments. Here, we present a rare case of hematogenous osteosynthesis-associated infection involving *Shewanella algae* affecting an 18-year-old patient who was operated on for 12 ^th^ thoracic vertebrae and 4th lumbar vertebrae fractures occurring in an aquatic environment. We performed surgical debridement with subsequent double course parenteral antibiotherapy that was then adapted to bacteria sensitivities for three weeks. After a follow-up of six months, the patient had no signs of recurrent infection. The presence of infected dermabrasions and the concordance between germs isolated in operative samples and in blood cultures presumes that the contamination was hematogenous.

## Background

*Shewanella* is an aquatic Gram-negative bacillus and is widely found throughout the environment. The most commonly reported clinical presentation is skin and soft tissue infection, ^1^^–^^3^ often preceded by exposure to seawater. ^4^ Bacteremia is often found in premature neonates with congenital pneumonia, patients with infections of the soft tissues of the lower limbs and with underlying health issues such as chemical esophagitis, cholangitis and liver abscess. ^5^ All the cases of osteosynthesis-associated infection involving *Shewanella* reported in the literature occurred after a direct contamination following open fractures, most often in aquatic environments. ^6^^–^^9^ We report the first case of osteosynthesis-associated infection caused by *Shewanella algae* via haemathogenic route.

## Case presentation

**Figure 1.**
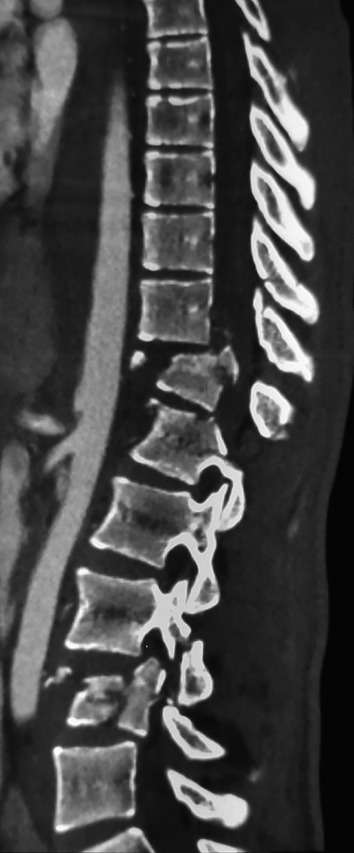
CT scan showing a burst fracture of 12 ^th^ thoracic vertebra and the 4 ^th^ lumbar vertebra.

An 18-year-old patient with no previous medical history of note was admitted to the intensive care unit after he fell into a well resulting in polytrauma. In addition to head and thoracic injuries, the whole body CT revealed a burst fracture of 12 ^th^ thoracic vertebra with section of the spinal cord and complete paraplegia, burst fracture of 4 ^th^ lumbar vertebra ( Figure 1). Both fractures were closed. In cutaneous clinical examination we found multiple water-soiled dermabrasions in both legs.

**Figure 2.**
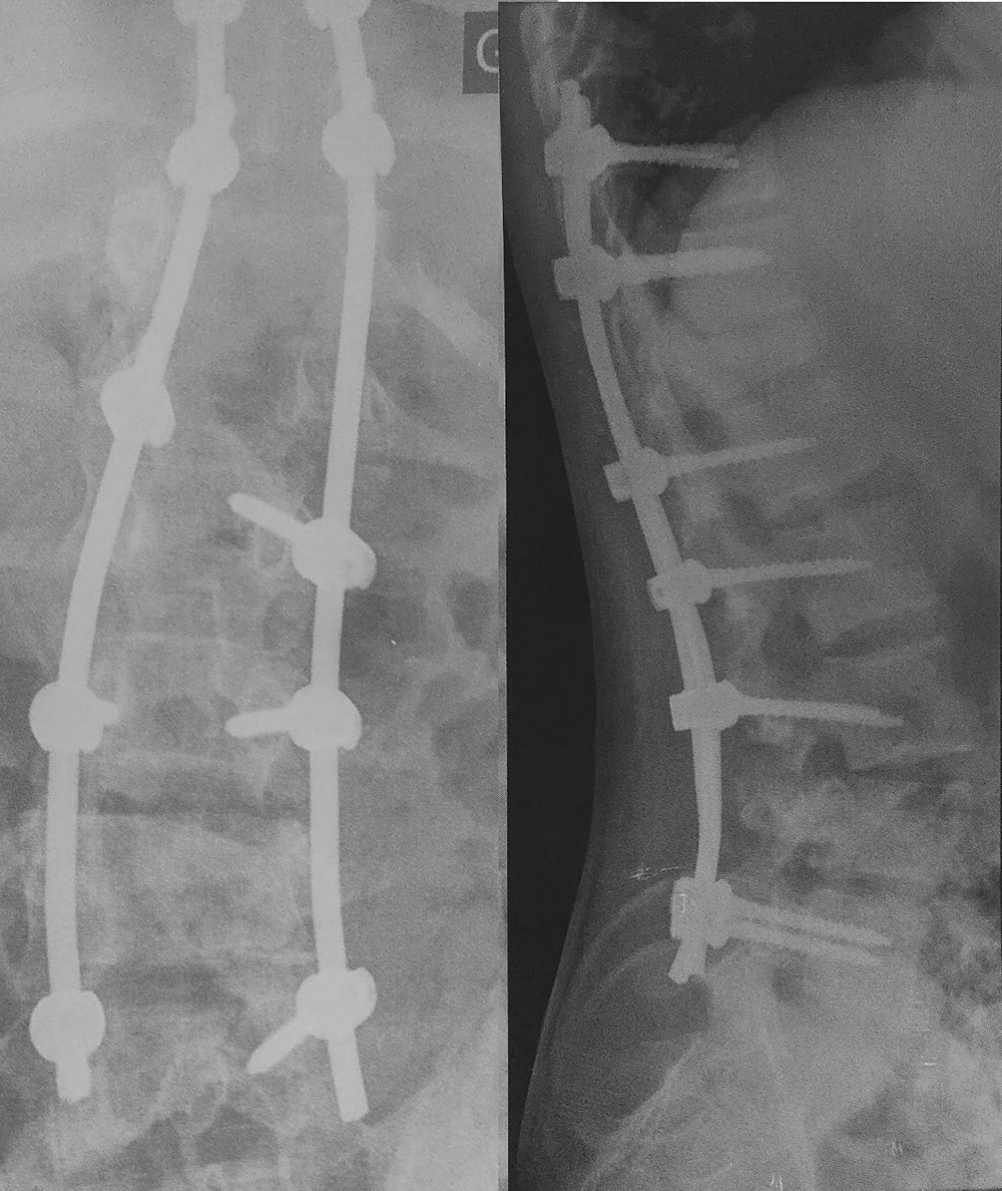
Postoperative anteroposterior and profile radiography of the T10-L5 postero-lateral fusion.

He was operated on in the orthopaedic surgery department, and postero-lateral fusion was performed from the 10 ^th^ thoracic vertebra to the 5 ^th^ lumbar vertebra ( Figure 2).

**Figure 3.**
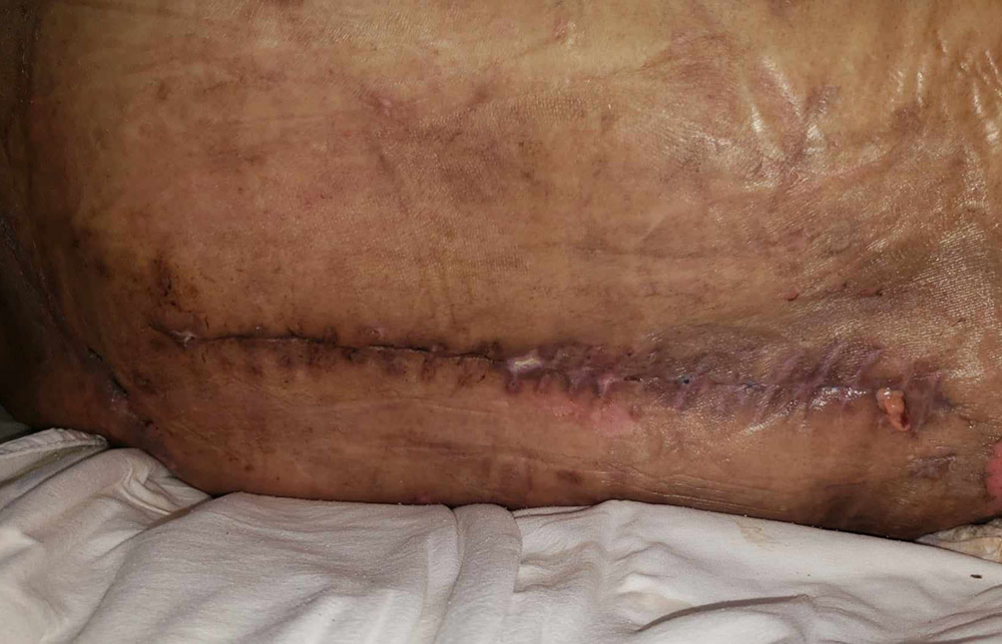
A clinical photograph of the surgical wound showing inflammatory signs with serous discharge.

At the 10 ^th^ post-operative day the patient presented fever (39.5°C), redness and swelling around the surgical wound with serous discharge ( Figure 3). Dermabrasions in lower limbs were infected. The vital signs included blood pressure, 120/60 mm Hg (NR: ≥ 90/60 mm Hg); respiration, 20 breaths per minute (NR: 12–18 breaths per minute); pulse, 95 beats per minute (NR: 60–100 beats per minute). Investigations showed a high white cell count (17.6 × 10 ^9^/L) (NR: 4.5–11 × 10 ^9^/L) and a raised C-reactive protein (176 mg/L) (NR: <0.3 mg/L). Three blood cultures were performed.

The patient was reoperated on the 11 ^th^ post-operative day. Intraoperatively, we found abundant pus with infected necrotic tissues that were then cleaned and debrided. We took five deep bacteriological samples. The operative wound was closed on aspiratifs Redon drain. One of the blood cultures became positive, Gram staining performed from culture showed Gram-negative rods. They were identified as *Shewenella algae* by vitek 2. Intraoperative deep tissue specimens grew *Shewanella algae* and *Klebsiella pneumoniae.* *Shewanella algae* was resistant to amoxicillin, amoxicillin-clavulanic acid and levofloxacin, had intermediate susceptibility to trimethoprim-sulfamethoxazole and was sensitive to imipenem/cilastatin. *Klebsiella pneumoniae* was multi-resistant and was only sensitive to colistin. The patient had a double course of parenteral antibiotics (Imipenem/cilastatin at a dose of 500/500 mg/6 hours and colistin at a dose of 3 MUI/8 hours) for 25 days. The patient had minor adverse events such as epigastralgia and vomiting, which resolved with symptomatic treatment.

After three weeks of antibiotics, white cell count and C-reactive protein normalized. The surgical wound healed with no fistula. The patient was addressed to physical medicine and rehabilitation department. At eight months follow-up, the patient had no signs of recurrent infection.

## Discussion

*Shewanella* has been regarded as an uncommon source of human infection. Despite being identified more than 70 years ago, ^1^ our understanding of the bacterium’s spread and the symptoms it causes comes primarily from a restricted set of individual case studies. Predominantly concentrated in tropical regions, the highest frequency of occurrences is noted within Southeast Asia, Southern Europe, and Africa. ^10^ They naturally exist in various environments like water of all types, raw fish, oily food, and soils. ^2^^,^^5^ Human infections involve *Shewanella algae*, putrefaciens, halitosis, and xiamenensis. However, the more offending species are *Shewanella algae* and putrefaciens accounting for more than 80% of cases. ^1^ *Shewanella* infections can be serious leading to life-threatening conditions such as necrotizing fasciitis and septic shock. ^11^^–^^14^ The route of infection is more likely cutaneous (wounds, leg ulcers, etc.), and, less frequently hepatobiliary or respiratory. ^15^ Malignancy, hepatobiliary disease, diabetes, immunodepression, dysregulated iron metabolism and chronic infections of lower limb have been reported to be risk factors for developing a *Shewanella* infection. ^1^^,^^2^^,^^15^^–^^17^ Although the patient received routine preoperative antibioprophylaxis based on 2 g of cefazolin and had no medical history, he developed infection.

In this case, *Klebsiella pneumoniae* was co-isolated in deep bacteriological samples. In fact, *Shewanella algae* are frequently identified in polymicrobial infections and the most common bacterial strains co-isolated are Enterobacteriaceae and marine flora bacteria. ^2^

Cases of osteosynthesis-associated infection caused by *Shewanella* are rare. In our review of the literature, all cases were secondary to open fractures of lower limbs occurring in an aquatic environment. ^6^^,^^8^^,^^9^^,^^18^ To the best of our knowledge, this is the first case in which osteosynthesis implant contamination was secondary to bacteremia. *Shewanella algae* have a significant ability to haematogenous diffusion. Indeed, Vignier ^9^ and Yousfi ^19^ observed that bacteremia occurred in respectively 28% and 18% of the cases they studied. Mortality rates were respectively 13 and eight per cent. Bacteremia can lead to severe secondary infection including instances of epidural spinal abscess, purulent pericarditis, acute gastroenteritis accompanied by bloody diarrhea, and meningoencephalitis, as reported in various studies. ^20^^–^^23^ The concordance between germs isolated in operative samples and in blood cultures presumes that the contamination was haematogenous, probably originating from infected dermabrasions in both legs.

As in other cases of osteosynthesis-associated infection reported in the literature, we performed surgical debridement with subsequent double course parenteral antibiotherapy that was then adapted to bacteria sensitivities. Colistin was selected because it was the only effective antibiotic against *Klebsiella pneumonae.* Imipenem/cilastatin was the only antibiotic available in the hospital to which *Shewanella* was sensitive. Typically, *Shewanella* displays susceptibility to erythromycin, fluoroquinolones, chloramphenicol, third and fourth generation cephalosporins, aminoglycosides, carbapenems, and to some degree, trimethoprim-sulfamethoxazole and tetracyclines. However, it exhibits resistance against first and second generation cephalosporins, penicillin, and colistin. ^24^ An emergence of resistance has been documented towards imipenem and piperacillin/tazobactam, which can be attributed to the presence of the class D beta-lactamase enzyme. ^23^ Hopefully, our microbial stain was sensitive to imipenem/cilastatin.

Currently, there are no established guidelines for the management of shewanella infections. However, certain reports have indicated that addressing *Shewanella* infections may necessitate a proactive approach involving both surgical debridement and administration of appropriate antimicrobial agents. This particular case underscores the importance of recognizing *Shewanella algae* as a potential offending pathogen in osteosynthesis-associated infection coming within the framework of secondary hematogenous infection even in patients without significant underlying medical conditions.

## Consent

Written informed consent for publication of clinical details and clinical images was obtained from the patient.

## Data Availability

All data underlying the results are available as part of the article and no additional source data are required.
