# Supplementary material for: Case Report: Shewanella algae, a rare cause of osteosynthesis-associated infection
Source: F1000Res. 2024 Sep 2;12:1465. Originally published 2023 Nov 13. [Version 2] doi: 10.12688/f1000research.142096.2 (PMC11375409; doi:10.12688/f1000research.142096.2)
Supplement: Peer review history

# Reviewer report: Reviewer response for version 2

**DOI:** 10.5256/f1000research.170594.r319866

**Published:** 2024-09-04

**Authors:** Elmostafa Benaissa

**Affiliations:** 1 Mohammed V Military Teaching Hospital, Rabat, Morocco

The authors have responded to my comments. I thank them for their efforts in responding to these comments.

Are enough details provided of any physical examination and diagnostic tests, treatment given and outcomes?

Partly

Is the case presented with sufficient detail to be useful for other practitioners?

Yes

Is sufficient discussion included of the importance of the findings and their relevance to future understanding of disease processes, diagnosis or treatment?

Yes

Is the background of the case’s history and progression described in sufficient detail?

Yes

Reviewer Expertise:

medical microbiologymolecular biologyinfectious diseases

I confirm that I have read this submission and believe that I have an appropriate level of expertise to confirm that it is of an acceptable scientific standard.

# Reviewer report: Reviewer response for version 1

**DOI:** 10.5256/f1000research.155595.r299831

**Published:** 2024-08-14

**Authors:** Elmostafa Benaissa

**Affiliations:** 1 Mohammed V Military Teaching Hospital, Rabat, Morocco

In this work by Masmoudi et al. a case of osteosynthesis-associated hematogenous infection involving the alga Shewanella is reported. The report is concise and addresses a case of infection by a relatively rare pathogen, but one for which there is a growing number of There have been only a few cases reported in the literature in recent years.

**General comments:**

 - Quality of presentation and structure of the manuscript: Satisfactory

 - To what extent are the conclusions supported by the data:Satisfactory

 - Do you have any concerns about possible image manipulation, plagiarism or any other unethical practice? : No

 - If this manuscript involves human and/or animal work, have the subjects been treated ethically and have the authors followed appropriate guidelines? : Yes

**specific comments:**

 - authors should specify in their report the method used to identify this bacterial species (maldi-tof or classical method...)

 - why did the authors speak of contamination via the blood-borne route, when the patient had open fractures exposed to aquatic, saprophytic and cutaneous flora? It may be that the osteosynthesis material was contaminated at the time of insertion and then infected, giving rise to bacteremia at the starting point of the osteosynthesis material.   clarification is required in this case.

Are enough details provided of any physical examination and diagnostic tests, treatment given and outcomes?

Partly

Is the case presented with sufficient detail to be useful for other practitioners?

Yes

Is sufficient discussion included of the importance of the findings and their relevance to future understanding of disease processes, diagnosis or treatment?

Yes

Is the background of the case’s history and progression described in sufficient detail?

Yes

Reviewer Expertise:

medical microbiologymolecular biologyinfectious diseases

I confirm that I have read this submission and believe that I have an appropriate level of expertise to confirm that it is of an acceptable scientific standard, however I have significant reservations, as outlined above.

# Response

**Published:** 2024-08-27

**Authors:** Sofiane Masmoudi

**Affiliations:** Orthopedic surgery, Rabta Hospital, Tunis, Tunis, Tunisia

Dear Dr Elmostafa,

 Thank you for reviewing our article. Your constructive comments made us aware of the lack of precision in certain aspects of our article. Indeed, it enabled us to give more details about microbiological techniques used to identify germs. Regarding your question about our theory of hemothogenic contamination, we have retained this theory given that the case involved a closed fracture of the thoracolumbar spine associated with water soiled dermabrations at a distance (on the both lower limbs) and the identification of shewanella algae on blood cultures. in this second version of the article, we have emphasized these elements in order to make the clinical case clearer for the reader.

# Reviewer report: Reviewer response for version 1

**DOI:** 10.5256/f1000research.155595.r272741

**Published:** 2024-06-11

**Authors:** Guillaume Beraud

**Affiliations:** 1 University Hospital of Poitiers, Poitiers, France

Masmoudi et al presents a case of *Shewanella algae* osteosynthesis-associated infection with the specificity that the pathogen was isolated in blood culture. They describe well the sequence that resulted in the infection, and their hypothesis is sound.

 English is good, the manuscript is well written and easy to read.

 I don't see any issue to be addressed.

Are enough details provided of any physical examination and diagnostic tests, treatment given and outcomes?

Yes

Is the case presented with sufficient detail to be useful for other practitioners?

Yes

Is sufficient discussion included of the importance of the findings and their relevance to future understanding of disease processes, diagnosis or treatment?

Yes

Is the background of the case’s history and progression described in sufficient detail?

Yes

Reviewer Expertise:

Infectious diseases

I confirm that I have read this submission and believe that I have an appropriate level of expertise to confirm that it is of an acceptable scientific standard.
